# A novel missense mutation in *GREB1L* identified in a three-generation family with renal hypodysplasia/aplasia-3

**DOI:** 10.1186/s13023-022-02553-w

**Published:** 2022-11-12

**Authors:** Sixian Wu, Xiang Wang, Siyu Dai, Guohui Zhang, Jiaojiao Zhou, Ying Shen

**Affiliations:** 1 Joint Laboratory of Reproductive Medicine, Gynaecology and Paediatric Diseases and Birth Defects of Ministry of Education, West China Second University Hospital, Sichuan University, Chengdu, 610041 People’s Republic of China; 2 Division of Ultrasound, West China Hospital of Sichuan University, Chengdu, 610041 People’s Republic of China

**Keywords:** *GREB1L*, RHDA3, RNA-seq, *PAX2*, *PTH1R*, WES

## Abstract

**Background:**

Renal hypodysplasia/aplasia-3 (RHDA3), as the most severe end of the spectrum of congenital anomalies of the kidney and urinary tract, is mainly caused by mutations in *GREB1L.* However, the mutations in *GREB1L* identified to date only explain a limited proportion of RHDA3 cases, and the mechanism of *GREB1L* mutations causing RHDA3 is unclear.

**Results:**

According to whole-exome sequencing, a three-generation family suffering from RHDA3 was investigated with a novel missense mutation in *GREB1L,* c.4507C>T*.* All three-generation patients suffered from unilateral absent kidney. This missense mutation resulted in sharp downregulation of mRNA and protein expression, which might lead to RHDA3. Mechanistically, through RNA*-*sequencing, it was found that the mRNA levels of *PAX2* and *PTH1R*, which are key molecules involved in the development of the kidney, were significantly downregulated by knocking out *GREB1L *in vitro*.*

**Conclusions:**

This novel missense mutation in *GREB1L* can be helpful in the genetic diagnosis of RHDA3, and the discovery of the potential mechanism that *GREB1L* mutations involved in RHDA3 pathogenesis can promote the adoption of optimal treatment measures and the development of personalized medicine directly targeting these effects.

**Supplementary Information:**

The online version contains supplementary material available at 10.1186/s13023-022-02553-w.

## Background

Congenital anomalies of the kidneys and urinary tract (CAKUT [MIM: 143400]) encompass a spectrum of developmental disorders of the urinary tract that range from mild vesicoureteral reflux to severe renal agenesis. These abnormalities can result in kidney damage and possibly renal failure [1]. Within the CAKUT phenotypic spectrum, renal hypodysplasia/aplasia (RHD [MIM: 191830]) is at the most severe end of the spectrum of CAKUT [2], affecting 0.5% of the general population [3]. Renal hypodysplasia/aplasia-3 (RHDA3) is an autosomal dominant disorder characterized by abnormal kidney development beginning in utero. To date, more than 75 genes have been implicated in the causation of isolated or syndromic forms of RHD and collectively account for 10%–15% of cases [3, 4]. Among the identified mutations, heterozygous mutations in *GREB1-*like retinoic acid receptor coactivator (*GREB1L*) were found in more than 40 probands with RHDA3 [3, 5–9]. However, not all the RHDA3 affected individuals can be explained by the reported *GREB1L* mutations.

*GREB1L* plays a major role in early metanephros and genital development [10]. Previous studies have shown that mutations in *GREB1L* are associated with bilateral renal hypoplasia, inner ear malformation, and deafness [10–12]. In many of the maternal cases with *GREB1L* mutations, their offspring were aborted or stillborn due to the severity of the malformations, such as bilateral renal aplasia [13]. CRISPR/Cas9 disruption or knockdown of *greb1l* in zebrafish has been reported to cause specific pronephric defects [3]. Other studies also proved that CRISPR/Cas9 mutagenesis of *Greb1l* in mice caused kidney agenesis phenotypes, implicating *Greb1l* involved in this disorder [2, 5]. However, the mechanism through which *GREB1L* regulates renal function remains unclear. Therefore, we do not know how *GREB1L* interacts with key regulators to manipulate the development of the kidneys.

In this study, we identified a novel missense mutation (c.4507C>T: p. R1503 W) of *GREB1L* in three patients suffering from RHDA3 in a three-generation family by whole-exome sequencing (WES). Bioinformatics methods were used to predict the pathogenicity of this missense mutation in *GREB1L.* Subsequently, it was found that the missense mutation led to the downregulation of mRNA and protein expression levels of *GREB1L* in HEK293T cells. Most importantly, after using shRNA to knock out *GREB1L* in HEK293T cells, the RNA-seq data suggested that *GREB1L* might reduce *PAX2* and *PTH1R* expression, which might be involved in the failure of kidney development.

## Results

### WES to identify the missense mutation c.4507C>T in ***GREB1L*** that causes RHDA3

**Fig. 1:**
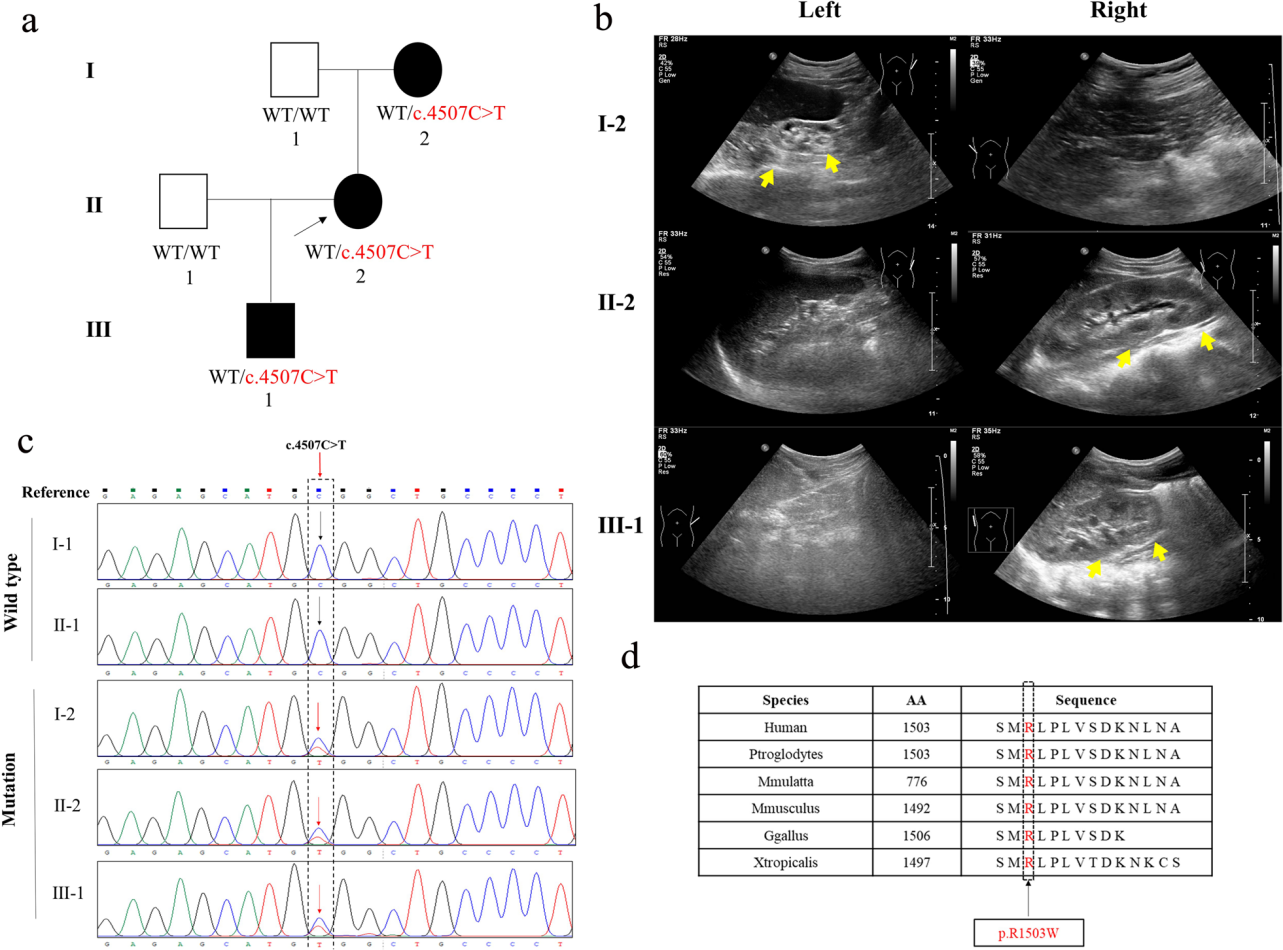
Clinical summary for the RHDA3 family. **a** Family pedigree. Cases presenting with unilateral kidney absence are in black. The black arrow denotes the proband. **b** Ultrasound pictures of patients’ bilateral kidneys. Yellow arrows denote the normal unilateral kidney, while the other unilateral kidney was absent. **c** Validation of the mutation *GREB1L*: NM_001142966: c.4507C>T in this family was performed by PCR sequencing. **d** Multiple sequence alignment of the mutation site for different species (black arrow denotes the position of the variant) (c.4507C>T: p.R1503W)

A three-generation Chinese family with RHDA3 was investigated in our study (Fig. 1a). The proband (II-2, 32 years old) was occasionally checked to have solitary kidney by ultrasound during first physical examination when she was eighteen years old (Fig. 1b), and her urine routine and renal function were normal during follow-up (Table 1). And then, she further completed the Computed Tomography (CT) examinations of chest, abdominal and pelvic to exclude the possibility of ectopic kidney. The proband’s mother (I-2, 55 years old), was diagnosed with right unilateral absent kidney (Fig. 1b) and she had chronic kidney disease secondary to left kidney dysplasia. The three-generation family had accepted ultrasound and the images were listed in Fig. 1b. However, all the family members refused to complete voiding cystourethrography or intravenous pyelography, considering the kidney damage caused by contrast medium. For the medical history presentation, the level of serum creatinine (Scr) in the proband’s mother was increased to 788 μmol/L (normal values: 48–79 μmol/L). She had been treated with regular peritoneal dialysis for seven years and then died of cardiovascular complications. The son of the proband (III-1, 3 years old) was found to have left solitary kidney during fetal life (Fig. 1b). The level of Scr and the level of estimated glomerular filtration rate (eGFR) in the son was normal (Table 1).

**Table 1:** Information on the clinical symptoms of the three-generation family

	Serum creatinine^a^ (Scr)	Estimated glomerular filtration rate^b^ (eGFR)	Urine routine and renal function
I-2	788 μmol/L (↑)	< 15 ml/min ·1.73 m^2^ (↓)	CKD^c^ stage 5
II-2	69 μmol/L	102.25 ml/min ·1.73 m^2^	Normal
III-1	44 μmol/L (↓)	177.36 ml/min ·1.73 m^2^ (↑)	Normal

^a^The normal reference range of serum creatinine is 48–79 μmol/L

^b^The normal reference range of the estimated glomerular filtration rate is 56–122 ml/min 1.73 m^2^

^c^*CKD* chronic kidney disease

By performing WES on the patients’ peripheral blood, we screened a missense mutation c.4507C>T in *GREB1L* based on the following criteria: (1) absent or exceedingly rare (MAF < 0.001) in population controls (ExAC, gnomAD, 1000 Genomes project, and In-house Chinese-Control); and (2) selection of SIFT [14], PolyPhen-2 [15] and M-CAP [16] to predict functional consequence. Meanwhile, we verified the missense mutation c.4507C>T again with PCR sequencing (Fig. 1c). The missense mutation c.4507C>T in *GREB1L* was absent in EXAC Browser, GnomAD, 1000 Genomes Project and In-house Chinese-Control (Table 2). The functional prediction of the missense mutation in SIFT, PolyPhen-2 and M-CAP was pathogenic (Table 2). According to the ACMG regulation, the mutation in *GREB1L* (NM_001142966: exon26: c.4507C>T: p.R1503W) was variant uncertain significance (VUS). Moreover, this variant site is highly conserved in many species according to Mutation Taster software (Fig. 1d). The scores of PhastCons and PhyloP indicated that this mutation site is highly conserved (Table 2). Therefore, the novel missense mutation c.4507C>T in *GREB1L* might be the genetic cause of RHDA3 in this family.

**Table 2:** Information on the missense mutation in the *GREB1L* gene

Gene	*GREB1L*	
cDNA mutation	NM_001142966: c.4507C>T	
Variant allele frequency	ExAC Browser	0
	GnomAD	0
	1000 Genomes Project	0
	In-house Chinese-Control	0
Amino acid sequence conservation	PhyloP	4.085
	PhastCons	1
Function prediction	SIFT	Deleterious
	PolyPhen-2	Probably damaging
	M-CAP	Possibly pathogenic

### The negative effects of the novel missense mutation on the ***GREB1L*** expression

**Fig. 2:**
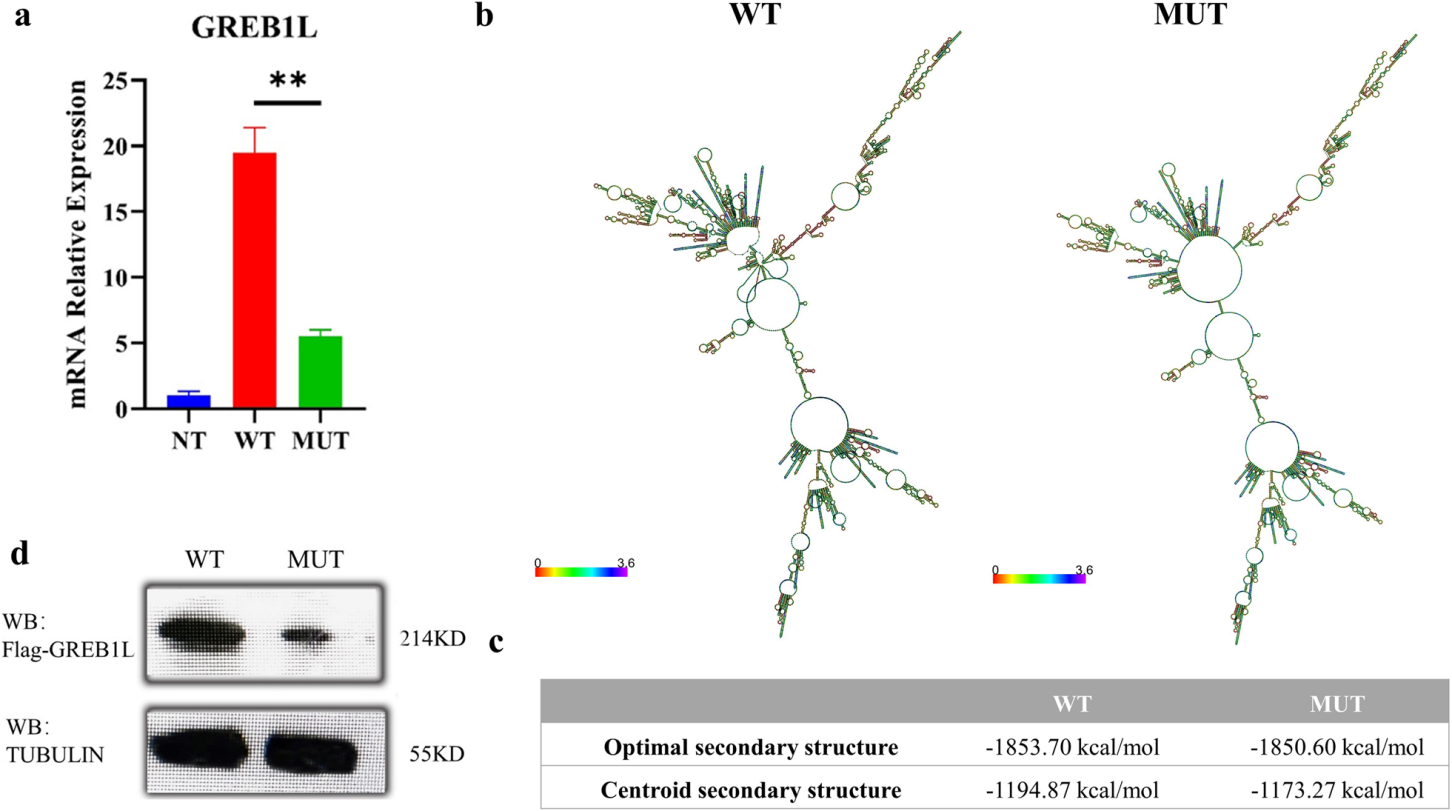
Expression analysis of the missense variant in *GREB1L*. **a** RT-PCR revealed downregulation of *GREB1L* mRNA expression caused by the mutation. **b** The centroid secondary structure of the WT and MUT *GREB1L* mRNA with RNAfold. **c** The prediction of minimum free energy of mRNA secondary structure. **d** Western blotting revealed downregulation of GREB1L protein expression caused by the mutation

We further constructed wild-type (WT-Flag-pc. DNA3.1-*GREB1L*) and mutant (MUT-Flag-pc. DNA3.1-*GREB1L*) plasmids to investigate the negative effects of *GREB1L* mutation on its expression. We found that *GREB1L* mRNA expression in HEK293T cells transfected with mutant plasmid was markedly diminished compared to those transfected with WT plasmid (Fig. 2a). To explore the reason for decreased mRNA expression, we predicted the change in the *GREB1L* mRNA structure caused by this missense mutation with RNAfold (http://rna.tbi.univie.ac.at/) [17–19]. There was no obvious difference in the optimal secondary structure between WT and MUT *GREB1L* (Additional file 1: Fig. S1), while notable changes were found in the centroid secondary structure of the mutant *GREB1L* mRNA (Fig. 2b). Specially, the minimum free energy of optimal secondary structure went up only from − 1853.70 to − 1850.60 kcal/mol, indicating the dispensable effect on its optimal secondary structure, while the minimum free energy of centroid secondary structure increased by 21.6 kcal/mol, suggesting the important influence on its centroid secondary structure (Fig. 2c). Therefore, the results indicated that the decreased mRNA structure stability might cause the down-regulation of *GREB1L* mRNA expression.

Moreover, we checked the potential change of GREB1L protein expression caused by the missense mutation. Noticeably, the diminished GREB1L amount was observed in cells transfected with mutant plasmid compared to those transfected with WT plasmid (Fig. 2d). Collectively, these findings indicated that the abolished mRNA structure stability might cause the down-regulation of *GREB1L* mRNA expression, and consequently lead to the reduced GREB1L protein level, which might result in RHDA3.

### ***GREB1L*** regulates the expression of ***PAX2*** and ***PTH1R*** during kidney development, as shown by ***RNA-seq*** analysis

**Fig. 3:**
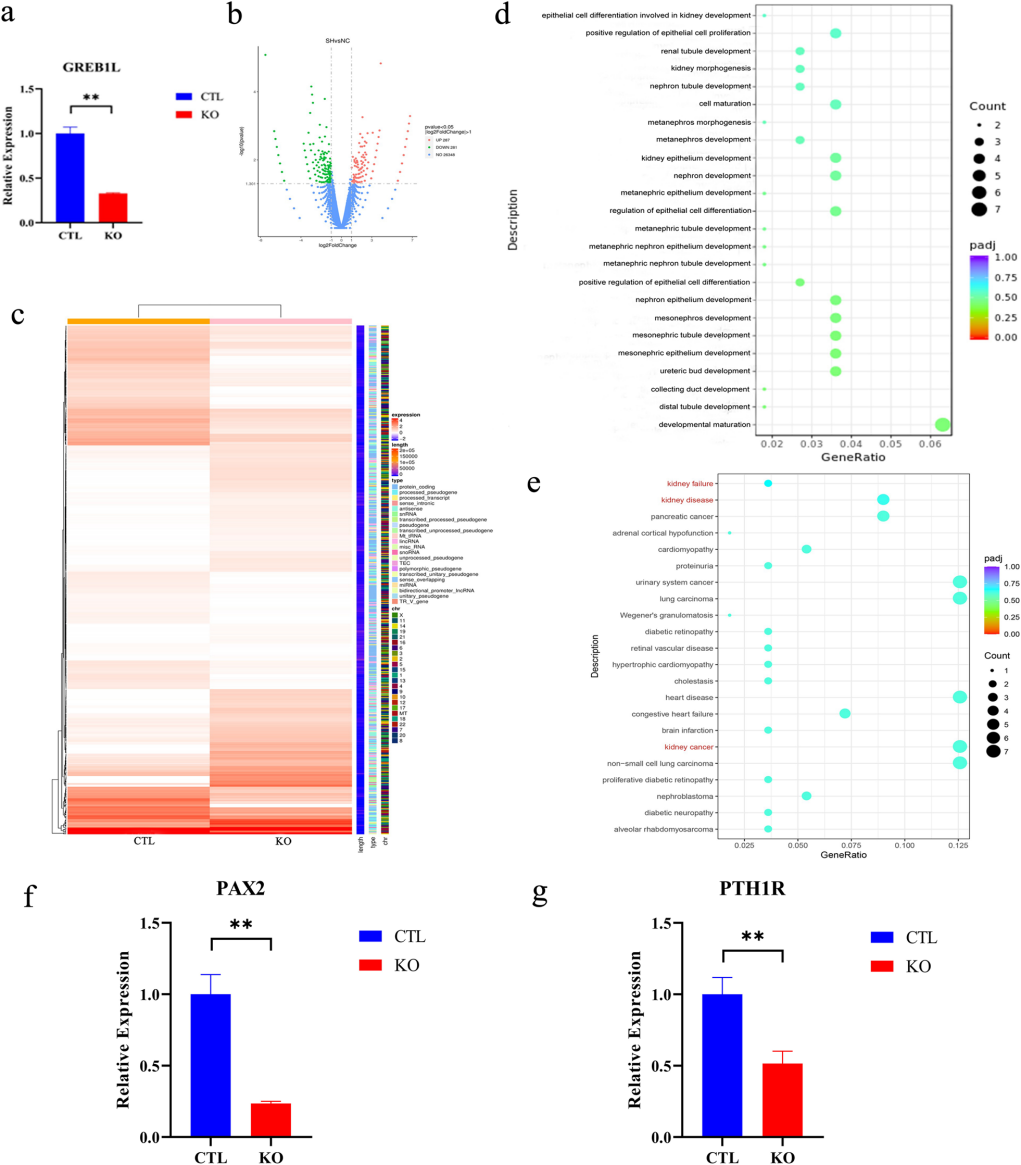
RNA-seq analysis of HEK293T cells with knock out of *GREB1L.* **a** Validation of the knockout efficiency of shRNA. **b** Volcano map of differentially expressed genes by RNA-seq of the knockout group (SH) and control group (NC). In total, 26,348 genes were quantified, of which 287 genes were upregulated and 281 genes were downregulated. **c** Heatmap of differentially expressed genes by RNA-seq of the knockout group (SH) and control group (NC). **d** Gene ontology (GO) terms related to downregulated genes of kidney development in the KO group (biological process). **e** Disease ontology (DO) terms related to downregulated genes. There were three pathways related to kidney disease, including kidney failure, kidney disease and kidney cancer. **f** and **g** The mRNA levels of *PAX2* and *PTH1R* in HEK293T cells with knock out of *GREB1L*. The results showed that after knocking out *GREB1L*, both *PAX2* and *PTH1R* mRNA expression significantly decreased, indicating that *GREB1L* could affect kidney development by regulating the expression of the *PAX2* and *PTH1R* genes

To further identify the key pathway by which *GREB1L* regulates kidney development, we constructed shRNA to knock out *GREB1L* in HEK293T cells, a cell line of human embryonic kidney cells. We verified the efficiency of shRNA (Fig. 3a). We then performed RNA-seq on HEK293T cells transfected with shRNA of *GREB1L* (KO cells) and control HEK293T cells (CTL cells). A total of 26,348 genes were quantified, of which 287 genes were upregulated and 281 genes were downregulated (Fig. 3b). The differentially expressed genes are shown in Fig. 3c. Gene ontology (GO) and disease ontology (DO) enrichment analyses were performed on the differentially expressed genes. According to the GO enrichment analysis, the downregulated genes after KO were mainly enriched in pathways related to kidney developmental maturation, such as distal tubule development, collecting duct development, ureteric bud development, mesonephric epithelium development, and metanephric nephron tubule development (Fig. 3d). DO enrichment analysis showed that the downregulated genes after KO were enriched in three pathways related to kidney disease: kidney failure, kidney disease and kidney cancer (Fig. 3e).

According to the literature, two of these differentially expressed genes, namely, paired box 2 (*PAX2*) [20, 21] [MIM: 167409] and parathyroid hormone 1 receptor (*PTH1R*) [8] [MIM: 168468], are directly related to kidney failure. *PAX2* is an essential transcription factor for kidney development. *Pax*2^+ / −^ mice develop kidney hypoplasia and vesicoureteral reflux3 [21]. The PTH1 receptor (PTH1R) is widely expressed in the kidney, where PTHrP exerts a modulatory action on renal function [22]. Therefore, we focused on these two genes, and the downregulation of *PAX2* (Fig. 3f) and *PTH1R* (Fig. 3g) expression was verified in KO cells compared with the CTL group. These results indicated that *GREB1L* might play a role in kidney development by regulating the expression of *PAX2* and *PTH1R*.

## Discussion

In this study, a three-generation family diagnosed with RHDA3 was examined and found to have a novel pathogenic missense mutation of *GREB1L*: c.4507C>T: p.R1503W, which results in the reduction both in *GREB1L* protein and mRNA levels. RNA-seq analysis showed that after *GREB1L* was knocked out, *PAX2* and *PTH1R* were downregulated, which suggested that *GREB1L* might regulate *PAX2* and *PTH1R* to affect the development of the kidney, leading to RHDA3. We found a novel missense mutation that expands the mutation spectrum of *GREB1L* and the potential mechanism through which it causes RHDA3, which provides new insights into the regulation of kidney development by *GREB1L*.

Renal hypodysplasia/aplasia, as the most severe end of the spectrum of CAKUT, has genetic heterogeneity. RHDA1 (OMIM: 191830) is caused by a mutation in the *ITGA8* gene (OMIM: 604063) on chromosome 10p13, and RHDA2 (OMIM: 615721) is caused by a mutation in the *FGF20* gene (OMIM: 605558) on chromosome 8p22. RHDA3 is caused by a mutation in *GREB1L* gene (OMIM: 617805) on chromosome 18q11. Frameshift mutations, missense mutations and splice site mutations are the main mutation types in *GREB1L* that have been reported to can cause RHDA3. Missense mutations are the most common type of mutations that cause RHAD3. Here, we also reported that the novel missense mutation c.4507C>T in *GREB1L*, which led to the degradation of *GREB1L* mRNA and protein. According to the prediction with RNAfold, the stability of mRNA secondary structure was reduced after mutating, which might lead to the decrease in mRNA expression, further causing the decrease in protein expression.

RHDA3 is caused by heterozygous *GREB1L* variants. However, the underlying mechanism of *GREB1L* regulating kidney development is still limited. Therefore, we chose to construct shRNA to knock out *GREB1L* in HEK293T cells, a cell line of human embryonic kidney cells. RNA-seq results revealed that knockout *GREB1L* reduced the expression of *PAX2* and *PTH1R*. *PTH1R* is widely expressed in the kidney, and the mutations in *PTH1R* have also been reported to be associated with kidney disease [23]. Human *PAX2* mutations have been associated with abnormalities in the developing and adult kidney [24]. We thus speculated that *GREB1L* mutations resulting in RHDA3 might be associated with the abnormal expression of *PAX2* and *PTH1R*.

In the previous reports, besides unilateral or bilateral renal agenesis, patients with RHDA3 caused by *GREB1L* mutations also had congenital hydronephrosis, ureter and bladder aplasia and vesicoureteral reflux in urinary system. *GREB1L* mutations have also been reported to affect reproductive system, such as agenesis of ovaries, uterus, streak gonads, and vagina. More seriously, *GREB1L* mutations might cause fetal malformation/death (Additional file 2: Table S1) [2, 5, 6, 25, 26]. However, in our study, the patients mainly suffered from unilateral absent kidney, but did not exhibit phenotypes associated with other systems, suggesting that there might be other genes regulating the diseases or patients with different genetic backgrounds.

## Conclusions

In summary, we identified a novel, pathogenic, heterozygous mutation of *GREB1L* in a Chinese three-generation family, and the findings enriched the variant spectra of the *GREB1L* gene and suggested that genetic analysis can play a key role in RHDA3 diagnosis and prognosis. Meanwhile, we reported that *GREB1L* is an important regulator of *PAX2* and *PTH1R*, which provided a mechanism by which *GREB1L* regulates kidney development.

## Materials and methods

### Subjects

Peripheral blood samples were obtained from the family after informed consent was signed by the proband and all family members. Informed consent was obtained from all subjects involved in the study. The study was conducted according to the guidelines of the Declaration of Helsinki. This experiment on human subjects was approved by the Ethical Review Board of West China Second University Hospital, Sichuan University. The number of Institutional Review Board is 040.

### Whole-exome sequencing (WES)

Genomic DNA was extracted from peripheral blood leukocytes using a whole blood DNA purification kit (TIANGENE). For WES, exons were captured from 1 μg genomic DNA using high-throughput sequencing detection technology for the whole exome provided by the manufacturer. The Verita Trekker® variant site detection system and Enliven® variant site annotation interpretation system independently developed by Berry Genomics were used to analyse the data. Functional annotation was performed through ANNOVAR, and data were filtered by public databases, such as ExAC, 1000 Genomes Project, In-house Chinese-Control and GnomAD.

Candidate pathogenic variants in the family members and their flanking intronic regions of *GREB1L* in the unrelated population were validated by Sanger sequencing. PCR amplification was performed with Dyad Polymerase (Bio-Rad Laboratories). DNA sequencing of PCR products was conducted on an ABI377A DNA sequencer (Applied Biosystems). The primers used for PCR were in Additional file 3: Table S2.

### Plasmid construction and cell transfection

The full-length cDNA of *GREB1L* was synthesized and separately cloned into pcDNA 3.1 3*Flag. The *GREB1L* plasmids were synthesized and cloned by WZ Biosciences Inc. All mutant plasmids of *GREB1L* were generated by the Fast Mutagenesis System of TransGen Biotech Co., Ltd. (Beijing, China). The primers used to construct the point mutation plasmid were in Additional file 3: Table S2.

The constructed plasmid was transformed into *E. coli*. Then, 10 μL of plasmid-containing bacterial solution and 20 μL of antibiotic ampicillin (50 mg/mL) were added to 20 mL of Luria–Bertani medium. The bacteria were placed on a shaker at 37 °C overnight. After shaking was complete, researchers extracted the plasmid from E. coli.

The HEK293T cell line was obtained from the American Type Culture Collection (ATCC, USA). HEK293T cells were cultured in 6-well cell culture plates and 100 mm cell culture dishes (WHB, China) with basic DMEM containing 10% foetal bovine serum (Gibco, USA) and 0.1% penicillin/streptomycin in a humidified incubator at 37 °C with 5% CO_2_. According to the experimental scheme, *GREB1L* plasmids were transfected into HEK293T cells for 36–48 h.

### Quantitative PCR

The total RNA of the cells was extracted using TRIzol reagent (Invitrogen) and was converted to cDNA using a Revert Aid First-Strand cDNA Synthesis Kit (ThermoFisher). Quantitative PCR was performed using SYBR Premix Ex Taq II (TaKaRa) on an iCycler RT–PCR Detection System (Bio-Rad Laboratories).

The ΔΔCT method was used for data analysis. All assays were finished in triplicate for all samples. The *GAPDH* gene was used as an internal control. The primers for real-time PCR are listed in Additional file 3: Table S2.

### Western blotting

The proteins were extracted using radioimmunoprecipitation assay (RIPA) buffer that contained a protease and phosphatase inhibitor cocktail (Roche). Twenty micrograms of the denatured proteins were separated on 10% SDS–polyacrylamide gels and transferred to a polyvinylidene difluoride (PVDF) membrane (Millipore) for immunoblotting analysis. After blocking with Tris-buffered saline/Tween-20 (TBST) containing 5% bovine serum albumin (BSA) for 1 h at room temperature, the membranes were then incubated with the corresponding primary antibodies, 1:50 anti-FLAG (HPA052219, Sigma–Aldrich) and 1:200 anti-β-tubulin (ZM-0439, ZSGB-BIO), at 4 °C. Samples were incubated overnight. The binding of the primary antibodies was visualized using horseradish peroxidase-conjugated goat anti-rabbit or anti-mouse IgG (1:10,000, ZSGB-BIO, China). The signal intensities were measured using ECL (1305702, Millipore Corporation, Billerica, USA) and image analysis software (ImageJ, NIH).

### ***GREB1L*** KO cells and RNA quantification

Short hairpin RNA (shRNA) targeting *GREB1L* was synthesized by WZ Biosciences Inc. HEK293T cells were transfected with shRNA plasmid for 48 h. After *GREB1L* was knocked out, the cells were collected for subsequent experiments. The cells were divided into an experimental group (shRNA plasmid with transfection to knock out *GREB1L*) and a control group (shRNA plasmid without transfection to knock out *GREB1L*). Total amounts and integrity of RNA were assessed using the RNA Nano 6000 Assay Kit of the Bioanalyzer 2100 system. The sequence of shRNA plasmid was in Additional file 3: Table S2.

### RNA-seq

After the RNA quality test was performed, RNA library construction and subsequent Illumina transcriptome sequencing were carried out. The amount of gene expression is expressed as FPKM (fragments per kb per million fragments). DEseq2 software (1.20.0) with the adjusted p value padj < 0.05 was used for the differential expression analysis between samples. GO enrichment analysis of differentially expressed genes was implemented by the cluster Profiler R package (3.8.1), in which gene length bias was corrected. GO terms with corrected P values less than 0.05 were considered significantly enriched by differentially expressed genes. The DO database describes the function of human genes and diseases. DO pathways with corrected P values less than 0.05 were considered significantly enriched by differentially expressed genes.

## Supplementary Information

**Additional file 1. Fig. S1**: The optimal secondary structure of the WT and MUT mRNA with RNAfold.

**Additional file 2. Table S1**: The differences of phenotype between our patient and those previously reported.

**Additional file 3. Table S2**: The primers in this study.

## Data Availability

The datasets used and/or analysed during the current study are available from the corresponding author on reasonable request. The transcriptomics data have been submitted to the SRA database. The number is PRJNA765796.

## Abbreviations

RHDA3: Renal hypodysplasia/aplasia-3
CAKUT: Congenital anomalies of the kidneys and urinary tract
GREB1L: GREB1-like retinoic acid receptor coactivator
Scr: Serum creatinine
eGFR: Estimated glomerular filtration rate
WES: Whole-exome sequencing
PAX2: Paired box 2
PTH1R: Parathyroid hormone 1 receptor
RNA-seq: RNA sequencing

## References

[1] Vivante A, Kleppa MJ, Schulz J, Kohl S, Sharma A, Chen J (2015). Mutations in TBX18 cause dominant urinary tract malformations via transcriptional dysregulation of ureter development. Am J Hum Genet.

[2] Brophy PD, Rasmussen M, Parida M, Bonde G, Darbro BW, Hong X (2017). A gene implicated in activation of retinoic acid receptor targets is a novel renal agenesis gene in humans. Genetics.

[3] Sanna-Cherchi S, Khan K, Westland R, Krithivasan P, Fievet L, Rasouly HM (2017). Exome-wide association study identifies GREB1L mutations in congenital kidney malformations. Am J Hum Genet.

[4] Arora V, Khan S, El-Hattab AW, Dua Puri R, Rocha ME, Merdzanic R (2021). Biallelic pathogenic GFRA1 variants cause autosomal recessive bilateral renal agenesis. J Am Soc Nephrol.

[5] De Tomasi L, David P, Humbert C, Silbermann F, Arrondel C, Tores F (2017). Mutations in GREB1L Cause Bilateral Kidney Agenesis in humans and mice. Am J Hum Genet.

[6] Jacquinet A, Boujemla B, Fasquelle C, Thiry J, Josse C, Lumaka A (2020). GREB1L variants in familial and sporadic hereditary urogenital adysplasia and Mayer–Rokitansky–Kuster–Hauser syndrome. Clin Genet.

[7] Wang A, Ji B, Wu F, Zhao X (2020). Clinical exome sequencing identifies a novel mutation of the GREB1L gene in a Chinese family with renal agenesis. Genet Test Mol Biomark.

[8] Romero M, Ortega A, Olea N, Arenas MI, Izquierdo A, Bover J (2013). Novel role of parathyroid hormone-related protein in the pathophysiology of the diabetic kidney: evidence from experimental and human diabetic nephropathy. J Diabetes Res.

[9] Mallett AJ, Quinlan C, Patel C, Fowles L, Crawford J, Gattas M (2019). Precision medicine diagnostics for rare kidney disease: Twitter as a tool in clinical genomic translation. Kidney Med.

[10] Boissel S, Fallet-Bianco C, Chitayat D, Kremer V, Nassif C, Rypens F (2018). Genomic study of severe fetal anomalies and discovery of GREB1L mutations in renal agenesis. Genet Med.

[11] Kari E, Llaci L, Go JL, Naymik M, Knowles JA, Leal SM (2020). Genes implicated in rare congenital inner ear and cochleovestibular nerve malformations. Ear Hear.

[12] Schrauwen I, Kari E, Mattox J, Llaci L, Smeeton J, Naymik M (2018). De novo variants in GREB1L are associated with non-syndromic inner ear malformations and deafness. Hum Genet.

[13] Schrauwen I, Liaqat K, Schatteman I, Bharadwaj T, Nasir A, Acharya A (2020). Autosomal dominantly inherited GREB1L variants in individuals with profound sensorineural hearing impairment. Genes.

[14] Kumar P, Henikoff S, Ng PC (2009). Predicting the effects of coding non-synonymous variants on protein function using the SIFT algorithm. Nat Protoc.

[15] Adzhubei I, Jordan DM, Sunyaev SR. Predicting functional effect of human missense mutations using PolyPhen-2. Curr Protoc Hum Genet. 2013;Chapter 7:Unit7.20. 10.1002/0471142905.hg0720s76 PMC4480630 23315928

[16] Jagadeesh KA, Wenger AM, Berger MJ, Guturu H, Stenson PD, Cooper DN (2016). M-CAP eliminates a majority of variants of uncertain significance in clinical exomes at high sensitivity. Nat Genet.

[17] Mathews DH, Disney MD, Childs JL, Schroeder SJ, Zuker M, Turner DH (2004). Incorporating chemical modification constraints into a dynamic programming algorithm for prediction of RNA secondary structure. Proc Natl Acad Sci USA.

[18] Gruber AR, Lorenz R, Bernhart SH, Neuböck R, Hofacker IL. The Vienna RNA websuite. Nucleic Acids Res. 2008;36(Web Server issue):W70–4. 10.1093/nar/gkn188 PMC2447809 18424795

[19] Lorenz R, Bernhart SH, Höner Zu Siederdissen C, Tafer H, Flamm C, Stadler PF, et al. ViennaRNA Package 2.0. Algorithms Mol Biol. 2011;6:26. 10.1186/1748-7188-6-26 PMC3319429 22115189

[20] Raffone A, Travaglino A, Saccone G, Mascolo M, Insabato L, Mollo A (2019). PAX2 in endometrial carcinogenesis and in differential diagnosis of endometrial hyperplasia: a systematic review and meta-analysis of diagnostic accuracy. Acta Obstet Gynecol Scand.

[21] Saifudeen Z, Liu J, Dipp S, Yao X, Li Y, McLaughlin N (2012). A p53-Pax2 pathway in kidney development: implications for nephrogenesis. PLoS ONE.

[22] Bosch RJ, Ortega A, Izquierdo A, Arribas I, Bover J, Esbrit P (2011). A transgenic mouse model for studying the role of the parathyroid hormone-related protein system in renal injury. J Biomed Biotechnol.

[23] Bastepe M, Raas-Rothschild A, Silver J, Weissman I, Wientroub S, Jüppner H (2004). A form of Jansen's metaphyseal chondrodysplasia with limited metabolic and skeletal abnormalities is caused by a novel activating parathyroid hormone (PTH)/PTH-related peptide receptor mutation. J Clin Endocrinol Metab.

[24] Harshman LA, Brophy PD (2012). PAX2 in human kidney malformations and disease. Pediatric Nephrol.

[25] Sanna-Cherchi S, Khan K, Westland R, Krithivasan P, Fievet L, Rasouly HM (2017). Exome-wide association study identifies GREB1L mutations in congenital kidney malformations. Am J Hum Genet.

[26] Herlin MK, Le VQ, Hojland AT, Ernst A, Okkels H, Petersen AC (2019). Whole-exome sequencing identifies a GREB1L variant in a three-generation family with Mullerian and renal agenesis: a novel candidate gene in Mayer–Rokitansky–Kuster–Hauser (MRKH) syndrome. A case report. Hum Reprod.

