# Supplementary figures and images for: A novel missense mutation in GREB1L identified in a three-generation family with renal hypodysplasia/aplasia-3
Source: Orphanet J Rare Dis. 2022 Nov 12;17:413. doi: 10.1186/s13023-022-02553-w (PMC9652819; doi:10.1186/s13023-022-02553-w)

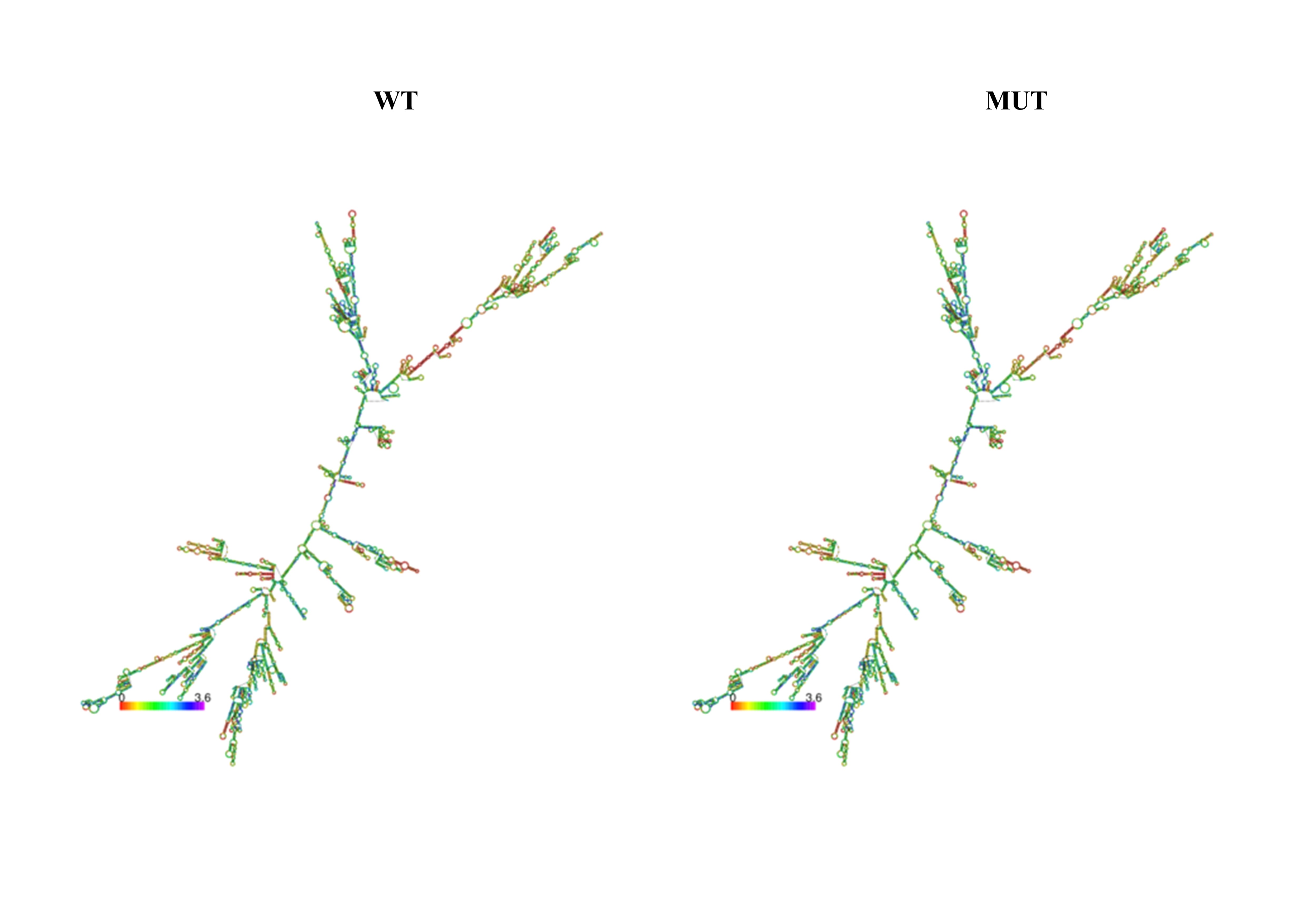

Supplement: Supplementary file 1 — Additional file 1. Fig. S1: The optimal secondary structure of the WT and MUT mRNA with RNAfold. [file 13023_2022_2553_MOESM1_ESM.jpg]
