# Supplementary material for: A novel missense mutation in GREB1L identified in a three-generation family with renal hypodysplasia/aplasia-3
Source: Orphanet J Rare Dis. 2022 Nov 12;17:413. doi: 10.1186/s13023-022-02553-w (PMC9652819; doi:10.1186/s13023-022-02553-w)
Supplement: Supplementary file 2 — Additional file 2. Table S1: The differences of phenotype between our patient and those previously reported. [file 13023_2022_2553_MOESM2_ESM.docx]

Supplement information

**Table S1. The differences of phenotype between our patient and those previously reported**

| Study | Renal hypodysplasia/aplasia | Abnormal development of bladders | Abnormal development of ureters | Abnormal development of uteruses | Abnormal development of ovaries | Fetal malformation/death |
| --- | --- | --- | --- | --- | --- | --- |
| Our cases | √ |  |  |  |  |  |
| Brophy et al. (2017) | √ |  |  |  |  | √ |
| Sanna-Cherchi et al. (2017) | √ | √ | √ | √ | √ |  |
| De Tomasi et al. (2017) | √ | √ | √ | √ |  | √ |
| Herlin et al. (2019) | √ | √ | √ | √ | √ | √ |
| Jacquinet et al. (2020) | √ | √ | √ | √ |  |  |
