# Supplementary material for: A novel missense mutation in GREB1L identified in a three-generation family with renal hypodysplasia/aplasia-3
Source: Orphanet J Rare Dis. 2022 Nov 12;17:413. doi: 10.1186/s13023-022-02553-w (PMC9652819; doi:10.1186/s13023-022-02553-w)
Supplement: Supplementary file 3 — Additional file 3. Table S2: The primers in this study. [file 13023_2022_2553_MOESM3_ESM.docx]

Supplement information

**Table S2.** The primers in this study

| ***Methods*** | ***Name*** | ***Forward primer*** | ***Reverse primer*** |
| --- | --- | --- | --- |
| *RT-PCR* | *GREB1L* | 5′ GTACTGGCCCAACCACATCA 3′ | 5′ CCAGGCGGTTTCTCTCCAAT 3′ |
|  | *GADPH* | 5′ ATGTTCGTCATGGGTGTGAA 3′ | 5′ GTCTTCTGGGTGGCAGTGAT 3′ |
|  | *PTH1R* | 5′GATCATCCAGGTGCCCATCC 3′ | 5′ GGATTTGAGCAGCTTCCGGT 3′ |
|  | *PAX2* | 5′ CATCCGGACCAAAGTTCAGCA 3′ | 5′CTATGGCTACAGTAGCACCAAG 3′ |
| *PCR* | *GREB1L* | 5′ AGGAGTGGCCTGGAAGTTTG 3′ | 5′ GAACTAGGTCTGGTGCCTGC 3′ |
| Construct the point mutation plasmid | *MUT-Flag-pc. DNA3.1-GREB1L* | 5′GGCAAGCACCTGGAGAGCATGTGGCTGCCCCT3′ | 5′ACATGCTCTCCAGGTGCTTGCCCTGCTTGCTG3′ |
| GREB1L KO Cells | *shGREB1L* | 5'CCGGGCTCTCCACAACTCCATAGAACTCGAGTTCTATGGAGTTGTGGAGAGCTTTTTG3' | |
|  | NCshRNA | 5'TTCTCCGAACGTGTCACGTTTCAAGAGAACGTGACACGTTCGGAGAATTTTTT3' | |
